# Relationship between Serum Indoxyl Sulfate and Klotho Protein and Vascular Calcification in Patients with Chronic Kidney Disease Stages 3–5

**DOI:** 10.1155/2024/8229604

**Published:** 2024-02-14

**Authors:** Yan Gao, Cong-Juan Zhao, Qiang Liu, Chen-chen Li, Zhe Li, Jing Li, Qian Wang, Li Zhang

**Affiliations:** ^1^Division of Nephrology, Affiliated Hospital of Hebei University, No. 212 of Yuhua East Road, Lianchi District, Baoding, Hebei 071000, China; ^2^College of Clinical Medicine, Hebei University, Baoding, China; ^3^Key Laboratory of Bone Metabolism and Physiology in Chronic Kidney Disease of Hebei Province, No. 212 of Yuhua East Road, Lianchi District, Baoding, Hebei 071000, China

## Abstract

**Objective:**

This study aims to explore the relationships between serum indoxyl sulfate (IS) and Klotho protein levels with vascular calcification in patients with chronic kidney disease (CKD) stages 3–5.

**Methods:**

From December 2021 to January 2023, a total of 108 CKD patients in stages 3–5 were enrolled in this cross-sectional investigation. Demographic information and routine clinical biochemistry test results were gathered. Serum levels of IS and Klotho were quantified through enzyme-linked immunosorbent assays. Furthermore, multislice spiral computed tomography was employed to evaluate vascular calcification. The association between serum IS or Klotho levels and abdominal aorta calcification was assessed using univariate analysis and logistic regression analyses.

**Results:**

With the progression of CKD stages, serum creatinine, phosphorus, intact parathyroid hormone (iPTH), serum IS, and abdominal aortic calcification exhibited incremental trends, while serum calcium and Klotho protein levels showed a diminishing trend, with statistically significant differences (*P* < 0.05). Significant differences were observed in age, blood phosphorus, calcium, total parathyroid hormone, serum IS, and Klotho protein levels between patients with and without aortic calcification (*P* < 0.05). Logistic regression analysis demonstrated that advanced age, high IS level, and low Klotho protein level were independent risk factors for abdominal aortic calcification in CKD patients (*P* < 0.05).

**Conclusion:**

This study indicates elevated serum IS levels and decreased Klotho protein levels in CKD patients. High IS level and low Klotho level were independent risk factors for abdominal aortic calcification.

## 1. Introduction

Chronic kidney disease (CKD) has become one of the international public health problems that seriously threaten human health. With kidney function progressively diminishing, CKD eventually develops into end-stage renal disease (ESRD). Cardiovascular disease (CVD) is the most common complication in patients with CKD and ESRD and the leading cause of death in patients with ESRD. Vascular calcification and its severity have long been recognized as important factors in the development of cardiovascular disease (CVD) in patients with CKD. The etiology of vascular calcification in CKD is multifactorial, influenced by both conventional and nontraditional elements including malnutrition, smoking, age, diabetes, uremic toxin accumulation, oxidative stress, and inflammation [1].

Indoxyl sulfate (IS) has garnered significant attention as one of the intestinal protein-binding uremic toxins in recent years. Current studies have shown that [1, 2] IS can upregulate the expression of osteoblast-specific proteins such as runt-related transcription factor 2 and osteopontin, thus promoting calcification. Klotho protein, a type I single-channel transmembrane protein, is highly expressed in the kidney. In a mouse model of chronic kidney disease-mineral bone disorder, the release of soluble Klotho alleviates hyperphosphatemia and vascular calcification [3]. In the late stage of CKD, the diminishing levels of Klotho protein compromise the effective regulation of bone minerals by fibroblast growth factor 23 (FGF23), thus accelerating the occurrence and development of vascular calcification in CKD [4]. At present, the mechanism underlying vascular calcification is not clear, and effective therapeutic strategies are lacking. Consequently, this study aims to investigate the correlation between serum IS level, Klotho protein levels, and vascular calcification in patients with CKD stages 3–5, understand the mechanisms underlying vascular calcification in CKD, and explore potential therapeutic targets.

## 2. Methods

### 2.1. Participants

A total of 108 patients with CKD stages 3–5 admitted to the Department of Nephrology of our hospital from December 2021 to January 2023 were selected. The inclusion criteria of CKD patients were as follows: (1) CKD stage from 3 to 5 according to the criteria for diagnosis and staging of CKD formulated in the K/DOQI guidelines; (2) age between 18 and 85 years; (3) medical history >6 months; and (4) no administration of phosphate binders or active vitamin D3 before admission. Exclusion criteria were as follows: (1) had previously received kidney transplantation or parathyroidectomy; (2) connective tissue diseases, blood diseases, malignant tumors, severe malnutrition, mental illness, or who are unable to cooperate with the examination for other reasons; (3) long-term use of hormones, immunosuppressants, and antibiotics; (4) severe infection, liver function damage, and cardiovascular and cerebrovascular accidents; (5) sarcomatoid nodular disease, HIV, and other diseases affecting the calcification status and soft tissue calcification of patients; and (6) pregnant or lactating patients.

All patients were divided into CKD stages 3-4 group (36 cases), CKD stage 5 nondialysis group (38 cases), and CKD stage 5 dialysis group (34 cases). Patients in the CKD stage 5 dialysis group were treated with stable hemodialysis with 3 h each time, 3 times per week, and the total duration of dialysis was 3–6 months. This short duration of dialysis can greatly weaken the effect of dialysis procedures on the degree of vascular calcification in patients [5]. All patients showed good responsiveness and sensitivity to dialysis treatment.

Twenty healthy subjects who underwent physical examination in the physical examination center of our hospital were selected as the healthy control group. Hypertension, diabetes, kidney disease, coronary heart disease, and other diseases were excluded. This study was approved by the Ethics Committee of our hospital (Ethics Committee approval no. HDFY-LL-2020-068). All participants signed the written informed consent.

### 2.2. General Information

Demographic data for all enrolled patients were meticulously documented, including gender, age, height, body mass index (BMI), smoking history, alcohol consumption history, and other pertinent variables. Detailed etiological records of CKD, including conditions such as diabetic nephropathy, chronic glomerulonephritis, and polycystic kidney disease, were compiled. In addition, histories of concurrent ailments such as diabetes, hypertension, coronary heart disease, and recent medication usage were thoroughly documented.

### 2.3. Laboratory Tests

Fasting blood samples were procured in the early morning to ensure consistency. For patients undergoing stable hemodialysis, blood samples were obtained before the last hemodialysis session of the week [6]. Levels of serum albumin, creatinine (SCr), glucose, calcium, phosphorus, and C-reactive protein (CRP) were measured using standard autoanalyzer techniques (Beckman DxC800). The iPTH levels were measured by electrochemiluminescence immunoassay (Roche Diagnostics GmbH, Mannheim, Germany). Serum IS and Klotho levels were measured by using an enzyme-linked immunosorbent assay (ELISA) method (Biomedica Co., Vienna, Austria).

### 2.4. Multislice Spiral Computed Tomography

The extent of abdominal aortic calcification in CKD patients was evaluated through multislice spiral computed tomography (abdominal CT). This assessment was conducted independently by two blinded researchers. The scoring criteria were established as follows: grade 0: the absence of abdominal aortic calcification; grade 1: calcification occupying less than 1/3 of the abdominal aortic circumference; grade 2: the maximum observed area of calcification is greater than 1/3 and less than 1/2 of the abdominal aortic circumference; and grade 3: the maximum observed area of calcification on abdominal CT scan was >2/3 of the abdominal aortic circumference. Based on the abdominal aortic calcification score, CKD patients were divided into noncalcification group (score 0) and calcification group (score 1–4).

### 2.5. Statistical Analysis

Statistical analysis was conducted using SPSS version 26.0 software. Normally distributed measurement data were expressed as mean ± standard deviation ($\overset{¯}{x}$ ± *s*). Independent sample *t*-test or one-way analysis of variance was used for intergroup comparison. The data without normal distribution were represented by median and quartile (Q1–Q3), and intergroup comparisons were performed by using the nonparametric Kruskal–Wallis test. Counting data are expressed as a number of cases (percentage) (cases (%)), and the *χ*^2^ test is used for intergroup comparison. The risk factors of abdominal aortic calcification were analyzed by binary logistic regression analysis. When *P* < 0.05, the difference was statistically significant.

## 3. Results

### 3.1. Comparison of Clinical and Biological Characteristics

Twenty healthy controls were enrolled, with an average age of 49.50 ± 12.31 years and an average BMI of 26.79 ± 4.38 kg/m^2^. All participants in the control group had no hypertension, diabetes, cardiovascular disease, or abdominal aortic calcification. All indicators are within the normal range (Table 1). A total of 108 patients with CKD, including 58 males (53.7%) and 50 females (46.3%), with an average age of 55.63 ± 13.51 years and an average BMI of 24.72 ± 3.57 kg/m^2^ were selected. The primary nephritis was chronic glomerulonephritis in 71 cases (65.74%), diabetic nephropathy in 27 cases (25.00%), polycystic kidney disease in 5 cases (4.63%), and other causes in 5 cases (4.63%). There were 44 cases with coronary heart disease (40.74%), 53 cases with diabetes (49.07%), and 100 cases with hypertension (92.59%), as shown in Table 1. There was no significant difference in age, albumin, hypertension, CRP, diabetes, and coronary heart disease between CKD stages 3-4, CKD stage 5 nondialysis, and CKD stage 5 dialysis patients (*P* > 0.05). With the progression of CKD, serum creatinine, serum phosphorus, iPTH, and abdominal aortic calcification gradually increased, while serum calcium levels showed a decreasing trend with statistical significance (*P* < 0.05), as shown in Table 1.

### 3.2. Comparison of Serum IS and Klotho Levels in CKD Patients

No statistically significant differences in age were observed among the groups (*P* > 0.05). However, when compared to the healthy control group, the CKD group exhibited higher serum IS levels and lower Klotho levels (*P* < 0.05). Furthermore, with decreasing renal function, the serum IS levels progressively increased, peaking in the CKD stage 5 dialysis group, while serum Klotho levels gradually declined, with a statistically significant difference (*P* < 0. 05), as shown in Table 2.

### 3.3. Univariate Analysis for Abdominal Aortic Calcification in CKD Patients

All CKD patients were divided into two groups: the abdominal aortic calcification group (75 cases, 69.44%) and the nonabdominal aortic calcification group (33 cases, 30.56%). Significant differences were observed between these two groups in terms of diabetes, coronary heart disease, age, serum phosphorus, serum calcium, and iPTH (*P* < 0.05). There was no significant difference in hypertension, body mass index, serum creatinine, and albumin (*P* > 0.05), as shown in Table 3. The abdominal aortic calcification rate of the CKD stages 3-4 group was 25.33%, and that of the CKD stage 5 group was 74.67%, with statistical significance (*P* < 0.01). In addition, the abdominal aortic calcification group had higher serum IS levels and lower Klotho levels than those in the nonabdominal aortic calcification group, and these differences were statistically significant (*P* < 0.001).

### 3.4. Multivariate Logistic Regression Analysis for Abdominal Aortic Calcification in CKD Patients

Based on the results of univariate analysis, variables that exhibited significant associations with aortic calcification were subjected to multivariate logistic regression analysis. These variables included diabetes, coronary heart disease, age, serum phosphorus, serum calcium, iPTH, serum IS, and Klotho. The logistic regression analysis revealed that age (OR: 1.295, 95% CI: 1.106–1.516, *P*=0.001) and IS (OR: 1.024, 95% CI: 1.006–1.043, *P*=0.009) had a positive impact on abdominal aortic calcification, while serum Klotho (OR: 0.002, 95% CI: 0.000–0.690, *P*=0.037) had a negative impact on abdominal aortic calcification. These results suggested that advanced age, high serum IS level, and low Klotho level were independent risk factors for abdominal aortic calcification, as shown in Table 4.

## 4. Discussion

Vascular calcification, characterized by abnormal deposition of calcium and phosphorus (hydroxyapatite crystals) on the vascular wall, is common in patients with atherosclerosis, diabetes, and kidney disease and even in very young dialysis patients [7]. Importantly, vascular calcification stands as an independent risk factor for cardiovascular events and mortality within the CKD patients. In this study, the incidence rate of vascular calcification was 69.44% (75/108). This high prevalence underscores the need for an in-depth exploration of the mechanism underlying vascular calcification and possible therapeutic targets to reduce the incidence of cardiovascular events and mortality in patients with CKD.

The mechanism of vascular calcification associated with CKD is very complex. Recent investigations have illuminated the complexity of this biological process, revealing its active involvement in multiple cellular signaling pathways and regulatory factors. The increase in serum phosphorus level is closely related to vascular calcification, but the effect of reducing serum phosphorus concentration by taking phosphorus binding agent is not good. Recent studies have shown that uremic toxin IS and kidney protective factor Klotho protein also play the key role in vascular calcification, which is expected to become a new target for the treatment of CKD-related vascular calcification.

IS is one of the enterogenic protein-binding uremia toxins that has been studied extensively in recent years. Lin et al. [8] found that serum IS levels gradually increased with the decline of renal function and reached a peak in patients with CKD stage 5 dialysis. Current studies have shown [9–11] that IS is related to renal tubule cell injury, renal tubule interstitial fibrosis, cardiac fibrosis, vascular calcification, and atherosclerosis. Klotho protein is a protein with powerful antiaging properties. It binds with fibroblast growth factor receptor 1 (FGFR1) and FGF23, which can play a role in regulating the synthesis of PTH and promoting phosphorus excretion [12]. Mice deficient in the Klotho gene developed high blood phosphorus and calcified arteries. Khodeir et al. [13] found that the serum Klotho level of CKD patients was significantly lower than that of healthy controls, and the serum Klotho content gradually decreased with the progression of CKD stages. It was found [14] that IS can induce CpG hypermethylation of the Klotho gene in smooth muscle cells, thereby decreasing the expression of the Klotho protein. Animal tests have also shown [15] that IS reduces the expression of Klotho protein in the kidney of hypertentic rats and promotes cell senescence, and IS can downregulate the expression of Klotho in proximal renal tubule cells by activating NF-*κ*B through the production of reactive oxygen species [16]. In this study, the serum IS concentration gradually increased and reached the highest level in maintenance dialysis patients, while the Klotho protein content gradually decreased, which was consistent with the abovementioned research results.

Several studies [2, 17] have shown that IS can promote vascular calcification in hypertensive rats. Opdebeeck et al. [18] observed increased aortic calcification in a rat model of adenine-induced CKD exposed to IS. Notably, higher IS levels were associated with greater vascular calcification in patients with end-stage kidney disease [19]. In the uremic environment, IS has also been found to induce CpG hypermethylation of the Klotho gene in smooth muscle cells, silencing these complementary calcification inhibitors and predisplacing them to vascular calcification [14]. Research has unveiled that mice with impaired Klotho gene expression exhibited blood vessel calcification, growth retardation, and osteoporosis. Hu et al. [20] reported that *α*-Klotho protein deficiency can cause vascular calcification in CKD mice. Zhang et al. [21] showed that soluble alpha-Klotho protein could inhibit calcification of human bone marrow mesenchymal stem cells. Chang et al. [22] reported that increasing *α*-Klotho protein expression through Intermedin1-53 could alleviate vascular calcification in CKD rats. This study indicated that old age, high level of IS, and low level of Klotho protein were independent risk factors for vascular calcification. In addition, the incidence of abdominal aortic calcification was higher in CKD stage 5 patients than that in CKD stages 3-4 patients, indicating a gradual increase in calcification incidence with CKD stage progression. This phenomenon may be attributed to the irreversible loss of renal function, CKD exacerbation, elevated serum IS concentrations, decreased Klotho protein levels, and disturbances in calcium, phosphorus, and PTH, ultimately culminating in vascular calcification.

In summary, the prevalence of vascular calcification is high in patients with CKD, and serum IS and Klotho protein levels are closely related to vascular calcification. The increase in serum IS level and the decrease in Klotho protein level are independent risk factors for vascular calcification and may become a new target for the treatment of vascular calcification in CKD.

## Figures and Tables

**Table 1:** Comparison of clinical data of CKD groups.

Group	Control group (20 cases)	CKD stages 3-4 (36 cases)	CKD stage 5 nondialysis (38 cases)	CKD stage 5 dialysis (34 cases)	*F/x* ^2^ */z*	^a^ * P* value
Age (years)	49.50 ± 12.31	57.03 ± 13.08	55.45 ± 14.69	54.35 ± 12.80	0.344	0.710
BMI (kg/m^2^)	26.79 ± 4.38	26.07 ± 3.46	24.96 ± 3.73	23.01 ± 2.84	7.329	0.001
Hypertension (cases (%))	0	32 (88.89%)	36 (94.74%)	32 (94.12%)	1.090	0.580
Diabetes (cases (%))	0	21 (58.33%)	16 (42.11%)	16 (47.06%)	2.029	0.363
Coronary heart disease (cases (%))	0	10 (27.78%)	19 (50.00%)	15 (44.11%)	4.016	0.134
CRP (mg/L)	4.54 ± 0.84	21.18 ± 57.24	20.00 ± 39.81	23.3 ± 25.81	0.048	0.953
Ca (mmol/L)	2.57 (2.48, 2.69)	2.28 (2.03, 2.43)	2.03 (1.96, 2.11)	1.84 (1.76, 2.02)	52.406	0.000^*∗*^
P (mmol/L)	1.24 (1.05, 1.53)	1.39 ± 0.27	2.51 ± 0.59	2.53 ± 0.37	79.442	0.000^*∗*^
iPTH (pg/mL)	33.54 (15.18, 59.32)	110.15 (74.58, 297.65)	478.55 (372.00, 648.43)	636.75 (374.23, 923.78)	26.999	0.000^*∗*^
ALB (g/L)	48.11 ± 6.85	31.78 ± 4.21	32.08 ± 4.49	30.24 ± 4.67	1.727	0.183
Cr (*µ*mol/L)	80.5 (48.1, 116.7)	231.5 (164.7, 282.2)	692.0 (561.0, 983.2)	703.0 (548.5, 956.2)	65.758	0.000^*∗*^
Abdominal aortic calcification (cases (%))	0	19 (52.78%)	26 (68.42%)	30 (88.24%)	10.389	0.006^*∗*^

^*∗*^ *P* < 0.05. CKD, chronic kidney disease; BMI, body mass index; CRP, C-reactive protein; iPTH, intact parathyroid hormone; ALB, albumin; Cr, creatinine. ^a^*P* value, between-group comparisons for the three CKD groups (excluding the control group).

**Table 2:** Comparison of age, IS, and Klotho protein among groups.

Group	Healthy control (20 cases)	CKD stages 3-4 (36 cases)	CKD stage 5 nondialysis (38 cases)	CKD stage 5 dialysis (34 cases)	*F/H*	*P* value
Age (years)	49.50 ± 12.31	57.03 ± 13.08	55.45 ± 14.69	54.35 ± 12.80	1.412	0.242
IS (pg/mL)	60.72 (53.94, 64.74)^abc^	120.06 (90.53, 222.45)^ab^	345.60 (269.10, 438.05)^a^	731.49 (562.47, 803.55)	98.49	0.000
Klotho (ng/mL)	1.30 (1.04, 1.37)^abc^	0.91 (0.58, 1.20)^ab^	0.59 (0.45, 0.82)^a^	0.42 (0.36, 0.52)	70.48	0.000

^a^Compared with CKD stage 5 dialysis group *P* < 0.05; ^b^compared with CKD stage 5 nondialysis group *P* < 0.05; ^c^compared with CKD stages 3-4 dialysis group *P* < 0.05. IS, indoxyl sulfate; CKD, chronic kidney disease.

**Table 3:** Analysis of clinical indexes in the group with and without abdominal aortic calcification.

Group	Nonabdominal aortic calcification (33 cases)	Abdominal aortic calcification (75 cases)	*t/x* ^2^ */z*	*P* value
Age (years)	44.09 ± 11.65	60.71 ± 10.93	−7.130	0.000^*∗*^
Male (cases (%))	23 (69.70%)	35 (46.67%)	4.889	0.027^*∗*^
BMI (kg/m^2^)	25.28 ± 4.23	24.46 ± 3.22	1.093	0.277
Hypertension (cases (%))	29 (87.88%)	71 (94.67%)	1.427	0.232
Diabetes (cases (%))	9 (27.27%)	44 (58.67%)	9.038	0.003^*∗*^
Coronary heart disease (cases (%))	6 (18.18%)	38 (50.67%)	10.017	0.002^*∗*^
Ca (mmol/L)	2.16 (2.04, 2.40)	1.99 (1.86, 2.05)	−5.872	0.000^*∗*^
P (mmol/L)	1.88 ± 0.68	2.26 ± 0.66	−2.748	0.007^*∗*^
iPTH (pg/mL)	145.00 (75.86, 431.65)	484.10 (189.90, 700.10)	−2.801	0.005^*∗*^
ALB (g/L)	30.85 ± 4.79	31.64 ± 4.36	−0.842	0.402
Cr (*µ*mol/L)	388.0 (207.5, 966.5)	584.0 (306.0, 758.0)	−0.764	0.445
CKD stage (cases (%))			7.069	0.008^*∗*^
Stages 3-4	17 (51.52%)	19 (25.33%)		
Stage 5	16 (48.48%)	56 (74.67%)		
IS (pg/mL)	109.62 (89.98, 196.34)	438.49 (261.48, 695.30)	−7.190	0.000^*∗*^
Klotho (ng/mL)	0.93 (0.85, 1.21)	0.47 (0.39, 0.58)	−7.558	0.000^*∗*^

^*∗*^ *P* < 0.05. BMI, body mass index; iPTH, intact parathyroid hormone; ALB, albumin; Cr, creatinine; CKD, chronic kidney disease; IS, indoxyl sulfate.

**Table 4:** Independent risk factor for abdominal aortic calcification.

Independent variable	*B*	SE	Wald	OR (95% CI)	*P* value
Age (years)	0.259	0.080	10.365	1.295 (1.106, 1.516)	0.001^*∗*^
IS (pg/mL)	0.024	0.009	6.812	1.024 (1.006, 1.043)	0.009^*∗*^
Klotho (ng/mL)	−6.320	3.035	4.336	0.002 (0.000, 0.690)	0.037^*∗*^

^*∗*^ *P* < 0.05. IS, indoxyl sulfate.

## Data Availability

All the data generated or analyzed during this study are included within this article.

## Additional Points

*What Is Already Known on This Topic?* Vascular calcification is a prevalent complication in cardiovascular disease among individuals with chronic kidney disease (CKD). Recent research has illuminated the contributions of indoxyl sulfate (IS) and Klotho to the initiation and development of vascular calcification. Nevertheless, the association between serum IS and Klotho levels and vascular calcification in patients at CKD stages 3–5 remains further unexplored. *What This Study Adds?* With the progression of CKD stages, serum IS exhibited incremental trends, while serum Klotho protein levels showed a diminishing trend. Patients with abdominal aortic calcification had higher serum IS and lower Klotho than those without aortic calcification. Moreover, the high IS level and low Klotho protein level were independent risk factors for abdominal aortic calcification in CKD patients. *How This Study Might Affect Research, Practice, or Policy?* Serum IS and Klotho protein exhibit a significant predictive value for disease progression and vascular calcification in CKD patients. The early detection of these biomarkers holds promise for improving prognosis and slowing down the development of vascular calcification in CKD patients. In addition, it opens new avenues for potential treatments targeting vascular calcification in CKD.
